# 

**Published:** 1884-09

**Authors:** 


### CONTENTS:

ART. I.—The Origin of Defective Enamel. By W. H. Eames, D. D. S.,............193

ART. II—The Septic Theory of Dental Caries By F. Searle, D D. S.,.............204

ART. III—The Chemistry of Dental Caries. By Wm. C. Wardlaw. D. D. S..........212

ART. IV.—Progress in Scientific and Artistic Dentistry. By W. E. Parsons........219

ART. V.—Articulation of Artificial Teeth for Their Better Retention.

By Dr. W. E. Driscoll..........227

### EDITORIAL, ETC.

A Dentist Interviewed..........231
Skin Grafting and Rhinoplasty..........234

### MONTHLY SUMMARY.

How to Make a Perfect Fitting Plate..........236

Antiseptic Absorbent Sponge..........239

Treatment of Epistaxis,..........240

Chloral Hydrate as a Vesicant..........240

